# Whole-genome analysis of mycobacteria from birds at the San Diego Zoo

**DOI:** 10.1371/journal.pone.0173464

**Published:** 2017-03-07

**Authors:** Wayne Pfeiffer, Josephine Braun, Jennifer Burchell, Carmel L. Witte, Bruce A. Rideout

**Affiliations:** 1 San Diego Supercomputer Center, University of California, San Diego, La Jolla, California, United States of America; 2 Wildlife Disease Laboratories, San Diego Zoo Global, San Diego, California, United States of America; St Petersburg Pasteur Institute, RUSSIAN FEDERATION

## Abstract

**Methods:**

Mycobacteria isolated from more than 100 birds diagnosed with avian mycobacteriosis at the San Diego Zoo and its Safari Park were cultured postmortem and had their whole genomes sequenced. Computational workflows were developed and applied to identify the mycobacterial species in each DNA sample, to find single-nucleotide polymorphisms (SNPs) between samples of the same species, to further differentiate SNPs between as many as three different genotypes within a single sample, and to identify which samples are closely clustered genomically.

**Results:**

Nine species of mycobacteria were found in 123 samples from 105 birds. The most common species were *Mycobacterium avium* and *Mycobacterium genavense*, which were in 49 and 48 birds, respectively. Most birds contained only a single mycobacterial species, but two birds contained a mixture of two species. The *M*. *avium* samples represent diverse strains of *M*. *avium avium* and *M*. *avium hominissuis*, with many pairs of samples differing by hundreds or thousands of SNPs across their common genome. By contrast, the *M*. *genavense* samples are much closer genomically; samples from 46 of 48 birds differ from each other by less than 110 SNPs. Some birds contained two, three, or even four genotypes of the same bacterial species. Such infections were found in 4 of 49 birds (8%) with *M*. *avium* and in 11 of 48 birds (23%) with *M*. *genavense*. Most were mixed infections, in which the bird was infected by multiple mycobacterial strains, but three infections with two genotypes differing by ≤ 10 SNPs were likely the result of within-host evolution. The samples from 31 birds with *M*. *avium* can be grouped into nine clusters within which any sample is ≤ 12 SNPs from at least one other sample in the cluster. Similarly, the samples from 40 birds with *M*. *genavense* can be grouped into ten such clusters. Information about these genomic clusters is being used in an ongoing, companion study of mycobacterial transmission to help inform management of bird collections.

## Introduction

Mycobacteriosis is of considerable concern in captive birds and is most commonly caused by *Mycobacterium avium* and *Mycobacterium genavense* [1]. For example, Hoop, Böttger, and Pfyffer [2] characterized mycobacterial infections among 48 pet birds in Switzerland and found that 71% were *M*. *genavense*, 17% were *M*. *avium-intracellulare* complex, and 12% were other mycobacterial species. Similarly, Witte, et al. [3], examined mycobacterial infections among 50 birds from the San Diego Zoo and found that 78% were *M*. *avium-intracellulare* complex and 22% were *M*. *genavense*. Both of these studies used conventional tests for species identification and combined *M*. *avium* and the closely related *M*. *intracellulare* as a complex.

Unraveling the transmission dynamics of mycobacteriosis requires that isolates (or samples) from different birds infected by the same species be differentiated to separate strains. Various DNA fingerprinting techniques have been used for such differentiation. For example, RADP and inverted-repeat typing separated strains of *M*. *avium* in farmed geese [4], RFLP separated strains of *M*. *avium* in captive water birds [5] and hens in an aviary [6], and AFLP and RADP identified *M*. *avium* and *M*. *intracellulare* strains in birds from the San Diego Zoo and its Safari Park [7]. The diversity of strains found in these studies suggest that apparent point source outbreaks may actually arise from different sources of infection, but the methods were not sufficient to elucidate transmission.

Whole-genome sequencing (WGS) allows two samples of the same species to be differentiated to the level of individual single-nucleotide polymorphisms (SNPs), which is not possible with DNA fingerprinting. In WGS studies of *M*. *tuberculosis* outbreaks in humans, Walker, et al. [8], inferred that transmission between hosts was likely only when samples from those hosts differed by 12 or fewer SNPs. By contrast, Niemann, et al. [9], showed that two samples with identical RFLP patterns differed by 130 SNPs. Numerous studies of *M*. *tuberculosis* have clearly shown the superiority of WGS for elucidating the fine-scale detail of transmission, for inferring the presence of mixed infections and within-host evolution, and for estimating mutation rates [8, 10–14]. A recent study of *M*. *avium* subsp. *paratuberculosis* in cattle also showed the advantages of WGS over DNA fingerprinting [15].

The study reported here is the largest ever performed on a single population of birds to completely characterize genetic relatedness of mycobacteria and the first to use WGS. The goals of the study were (1) to use WGS to catalog the diversity of mycobacteria found in a population of over 100 diseased birds from the San Diego Zoo and its Safari Park over a timespan of 24 years and (2) to identify genomic clusters of closely related mycobacteria. The results clearly demonstrate the value of using WGS for mycobacterial species identification and strain differentiation in birds.

## Materials and methods

### Selection of study population

The study population consists of a subset of birds diagnosed with avian mycobacteriosis at the San Diego Zoo and its Safari Park between 1992 and 2015. During this time period all birds housed in these facilities were under continuous observation by keepers and veterinarians. When a bird died it received complete postmortem examinations including histopathology performed on a full set of tissues by a board-certified veterinary pathologist. Avian mycobacteriosis was diagnosed in birds based on the presence of acid-fast bacilli in tissues (not including feces) using special stains (Ziehl-Neelson or Fite-Faraco) and was sometimes confirmed with culture by external laboratories or in-house at the Zoo’s Wildlife Disease Laboratories. Usually diagnoses were made postmortem, but occasionally clinical presentation permitted diagnosis of mycobacteriosis from tissue biopsies before a bird died or was euthanized with the disease.

The subset of birds further evaluated in this study was determined by searching the Zoo’s database of postmortem findings, limiting it to birds that were diagnosed with mycobacteriosis during the study period, that were not in quarantine, and that had either frozen tissue available for culture or viable isolates from previous cultures. In total, 167 birds met these criteria: 122 birds had either fresh or frozen tissues available for attempted culture in-house, and 45 had viable isolates previously retrieved from external labs.

### Isolation and culture of mycobacteria

During postmortem examination tissue samples were collected using aseptic techniques for subsequent mycobacterial culture. When acid-fast bacilli were present in multiple tissues, preference for culturing was given to tissues other than intestine to limit identification of pass-through organisms in fecal material. In some cases, multiple samples per bird, usually from different tissues, were cultured to check for reproducibility and increase the chances of successfully culturing mycobacteria. Culturing from fresh tissue was preferred, but often frozen tissues were used due to availability. An attempt was made to obtain sufficient DNA for analysis directly from tissue samples without culturing, but this was not successful as elaborated upon in the Discussion section.

For isolation of mycobacteria in external laboratories, fresh tissues were submitted directly for mycobacterial culture or frozen until submission. Tissues were sent to University of California San Diego Health System Clinical Laboratory (La Jolla, CA) or National Jewish Health Advanced Diagnostic Laboratories (Denver, CO) for mycobacterial culture. Isolated mycobacteria were received on slants and stored at 4°C for up to 13 years. Viable mycobacteria from these slants were subcultured for the present study. Utilizing aseptic techniques, a singular colony was isolated and inoculated into a Middlebrook 7H9 broth with glycerol (BD Biosciences, San Jose, CA). The broth was allowed to grow at 37°C and 5% CO_2_ under aerobic conditions for 4–6 weeks until confluent.

For mycobacterial culture in-house, fresh tissues were used unless the sample was previously frozen, in which case it was partially thawed on ice. Using a sterile Petri dish, approximately 10–25 mg of the tissue was cut and diced into smaller pieces using a sterile scalpel. The sample was then further homogenized in 10 ml of sterile molecular grade water within a Dounce homogenizer. The homogenized sample was transferred to a 50-ml conical tube containing 10 ml of MycoPrep™ Specimen Digestion/Decontamination reagent (BD Bioscience, Franklin Lakes, New Jersey) and processed according the manufacturer’s guidelines. Two Middlebrook 7H11 agar slants, two Mitchenson 7H11 selective agar slants, and two Lowenstein-Jensen slants (Edge Biologicals, Memphis, TN; BD Biosciences, San Jose, CA; Remel Microbiology Products, Lenexa, KS) were each inoculated with 100 μl of the sample sediment. One set of three slants (one of each type) was incubated at 37°C and one set at 30°C, both at 5% CO2 for up to 8–10 weeks.

If no colonies were visible after the maximum incubation time, the culture was considered negative. If colonies were visible then conventional PCR was performed to confirm mycobacteria as described next.

### Confirmation of mycobacteria by PCR

DNA was extracted from in-house cultures for subsequent mycobacterial genus confirmation prior to subculture. A singular colony was isolated from the culture slant utilizing a boiling and lysing DNA-extraction method as follows: Using aseptic techniques, a colony was picked and inoculated into a 2-ml screw cap tube containing 200 μl of molecular grade sterile water. The sample was heated to 100°C on a thermal block for 10 minutes. It was then removed from the block, vigorously vortexed, and allowed to cool at room temperature for 2 minutes. Next, the sample was centrifuged at maximum speed (~14,000 rpm) for 5 minutes to pellet the lysed culture. The supernatant containing the lysed mycobacterium culture DNA was carefully removed and transferred into a fresh tube.

PCR targeting one of three genes was then performed: a 439-bp 65-kDa heat shock protein sequence [16], a 1,030-bp 16S rRNA mycobacterium genus-specific sequence [17], or a 236-bp mycobacterium genus-specific dnaJ sequence [18].

### Whole-genome sequencing

DNA was extracted from all subcultures utilizing Qiagens QiaAMP DNA Mini Kit (Qiagen,Valencia, CA) following the manufacturer’s protocol with additional pretreatment steps to break down the waxy, tough phospholipid bilayer of the mycobacteria. The pretreatment steps were as follows: the aliquot of liquid culture was pelleted down by centrifuging the samples at 7500 RPM for 5 minutes at room temperature; the supernatant was discarded; and the pellet was treated with 180 μl of lysozyme solution (MP Biomedicals, Santa Ana, CA) for 1 hour at 37°C with a vortexing interval of 40 seconds at 30 and 60 minutes. DNA was quantified using the Qubit^R^ (Invitrogen, Waltham, MA). Samples with at least 1.0 μg or 0.3 μg of DNA were submitted to The Scripps Research Institute (TSRI; La Jolla, CA) for whole-genome sequencing using Illumina HiSeq 2000 or NextSeq 500 systems, respectively. Over the course of the study, a total of 132 samples from 113 birds were sequenced. S1 File provides information on the sequencing by sample number.

Most samples sequenced with the NextSeq 500 used the following, standard library preparation protocol: Extracted DNA (200 ng) was fragmented using a Covaris S2 (duty cycle = 10; intensity = 5; cycles per burst = 200 for two minutes) to generate fragments that were about 300-bp long. Fragmented DNA was prepared into sequencing libraries using the NEBNext® Ultra™ DNA Library Prep Kit for Illumina following manufacturer’s instructions. The library was PCR-amplified using Kappa HiFi polymerase with a 2X buffer for 6 cycles followed by a 1X Ampure XP bead cleanup. DNA products were then denatured in 0.1N NaOH and diluted to the appropriate concentration for running on the sequencing system.

Fourteen early samples sequenced on the NextSeq 500 used an alternate preparation protocol that often gave poorer assemblies than did the standard protocol. Five of these samples had sufficient DNA to regenerate libraries with the standard protocol. These samples were sequenced again, and the resequenced reads were used in subsequent analyses, while the reads from the alternate protocol were used for the other nine samples.

The first 52 samples were sequenced with a HiSeq 2000 to obtain 2x100-bp reads. As shown in the first sheet of S2 File, the average raw read coverage based upon the subsequent assembly varied from about 150x to nearly 1,000x, except for six samples with coverage less than 100x. Of the latter samples, myc41 and myc44 were sequenced twice, and myc42 was sequenced three times. The original and resequenced reads were then combined to increase the coverage, and the combined samples were designated myc41c, myc42c, and myc44c. Of these, the coverage of myc42c increased to above 150x, while that of myc41c and myc44c was still below 100x.

The remaining 80 samples were sequenced with a NextSeq 500 (due to discontinuation of the HiSeq 2000 at TSRI) to obtain reads that were either 2x150-bp long or of variable length up to 2x151-bp long. The raw read coverage based upon the subsequent assembly was similar to or somewhat higher than that for the HiSeq reads; nine samples had average raw read coverage above 1,000x. One sample–myc126 –was also sequenced twice, and the original and resequenced reads were combined and designated myc126c.

Twelve samples sequenced with the NextSeq were also sequenced with an Illumina MiSeq system at TSRI. Subsequent variant calling between the MiSeq reads and the corresponding NextSeq reads confirmed the reproducibility of the sequencing. No differences in high-confidence variants were found between the corresponding reads in the 12 samples.

HiSeq and NextSeq reads for 112 samples listed in the second sheet of S2 File were deposited in the NCBI Sequence Read Archive under Bioproject PRJNA351843. These are all samples found by WGS to contain mycobacteria excluding duplicates, which are repeat samples with the same genotype as another sample from the same bird. The duplicates are listed in the “Repeat genotypes” sheet of S2 File.

### Species identification based on WGS

#### Filtering the reads

Spot checks of the reads with FastQC 0.11.4 (http://www.bioinformatics.babraham.ac.uk/projects/fastqc) showed as usual that many of them had low quality scores near the right (3’) end. Also, many adapter sequences were present in the NextSeq reads.

Trimmomatic 0.32 (http://www.usadellab.org/cms/?page=trimmomatic) was used to remove the adapter sequences, if necessary, and to filter the reads to ensure high quality and to speed up the subsequent assemblies and variant calling. For each sample the two input files of paired reads were converted to four output files: two paired files where both reads survived the filtering and two unpaired files where one read survived but the other did not. The Trimmomatic parameters varied between samples to improve the assemblies and are listed in S1 File.

#### Assembling the reads

*De novo* assembly of the filtered reads for all samples was done with Velvet 1.2.10 (https://www.ebi.ac.uk/~zerbino/velvet). The k-mer value and the filtered reads that were used varied depending upon the sequencer and preparation protocol as noted in S1 File. The R2 unpaired reads were excluded for several NextSeq samples; those reads were few in number and of questionable quality according to FastQC, despite having been filtered.

Additional assemblies with other k-mer values confirmed that the values adopted were optimal or near optimal for maximizing the N50 length, which is a common criterion for determining the “best” assembly. Moreover, subsequent BLAST+ runs confirmed that identification of the species was relatively insensitive to the choice of k-mer.

The first sheet in S2 File l lists the assembly statistics for 131 of the 132 samples that were sequenced. The remaining sample–myc124 – had bad reads and could not be further analyzed.

Five samples had shorter than expected assembled lengths and are highlighted in yellow in the first sheet of S2 File. The reads for these sample–myc54a1, myc55a1, myc56a1, myc64a1, and myc126c –were reassembled with SPAdes 3.6.2 (http://bioinf.spbau.ru/spades) using multiple k-mer values as listed in S1 File, and the reassembled lengths increased to the expected values. All of these samples–four of which used the alternate preparation protocol–had read coverage that was less uniform across the genome than for the other samples.

Four samples with unusually long assemblies were subsequently found from the BLAST+ alignments described next to be a mix of multiple bacterial species, one of which was mycobacterial with a short matching length highlighted in yellow in the first sheet of S2 File. The reads for these samples–myc03, myc28, myc44c, and myc53 –were also reassembled with SPAdes, and the matching length of the mycobacterial species that was short increased in each case. For *M*. *avium* in myc53 the assembled and matching lengths increased dramatically, the latter from 37,574 bp to 4,919,324 bp, which is the entire genome. For *M*. *peregrinum* in myc28 the matching length increased by a more modest amount but still significant amount. For *M*. *avium* in myc03 and myc44c the matching lengths remained very short, presumably because most of the DNA in these samples was not mycobacterial.

Determining the species in a sample can be done with relatively low or nonuniform coverage. However, coverage sufficient for a good assembly is generally necessary for reliably calling variants.

#### Aligning the assemblies against the NCBI database

The assemblies were first aligned against the RefSeq database from NCBI (http://www.ncbi.nlm.nih.gov/refseq/about) as of March 17, 2016 using BLAST+ 2.2.29 (https://blast.ncbi.nlm.nih.gov/Blast.cgi?PAGE_TYPE=BlastDocs&DOC_TYPE=Download) with the "-max_target_seqs 1” option. To deal with the voluminous BLAST+ output, two custom Perl scripts, named identify-species and identify-strain, picked the best match for each contig and then output the total number of contigs and the matching length either by species or by strain above and below a specified identity threshold, which was taken to be 95%. Some samples contained multiple bacterial species. (These custom scripts and others mentioned by name herein are available at https://github.com/wpfeiffer/sequencescripts.)

The assembly for each sample was next aligned separately with BLAST+ against the closest matching strains using the “-best_hit_overhang 0.1 -best_hit_score_edge 0.1” options. Then another custom Perl script, named identity, was used to calculate the average nucleotide identity (ANI) over the total length of the matches, neglecting any matches below 90%. The best matching strain, ANI, and total matching length are listed for each sample in the first sheet of S2 File.

### Finding single nucleotide polymorphisms (SNPs) between samples of the same species

#### Variant calling

Variants between multiple samples of the same mycobacterial species, namely *M*. *avium*, *M*. *fortuitum*, *M*. *genavense*, and *M*. *intracellulare*, were called by two different codes: (1) GATK 3.5 (https://software.broadinstitute.org/gatk) using the HaplotypeCaller tool preceded by BWA, SAMtools, and Picard in accordance with the GATK best practices and (2) Cortex_var 1.0.5.20 (http://cortexassembler.sourceforge.net/index_cortex_var.html) together with its run_calls Perl script. The same filtered reads were used for variant calling as for assembly, even though Cortex_var initially filters or “cleans” the reads as well. Calling the variants with two different codes increased the confidence for the calls that agreed and provided guidance as to which code was better where the calls differed.

For GATK, HaplotypeCaller was used alone without GenotypeGVCFs since that generally gave a few more high-confidence variants than using both tools together. The default settings were adopted, except that “-ploidy 1” was set as appropriate for bacteria. The reference genomes used in the BWA read mapping were *Mycobacterium avium 104*, *M*. *fortuitum DSM 46621*, *M*. *genavense ATCC 51234*, and *M*. *intracellulare MOTT-64* from the NCBI database.

For Cortex_var the joint workflow with a CoordinatesOnly reference was used since it generally gave the same or a few more high-confidence variants than any of the other three workflows. The references adopted were the same as for BWA.

Since Cortex_var does assembly-assisted variant calling, one or more k-mer values had to be specified. Two k-mers were used– 33 and 63 –and the final output was the union of the variants from both k-mers. This allowed slightly more variants to be found than using only a single k-mer.

Cortex_var allows analyses with and without a population filter. Test runs showed that the SNPs passing the high-confidence filters described in the next subsection were the same with and without the population filter.

#### Filtering the variants to obtain high-confidence SNPs

Both GATK and Cortex_var generate many candidate, bi-allelic variants in a vcf file, but most of them are not of interest. Thus two custom Perl scripts were written to filter the vcf file to obtain high-confidence SNPs.

The first script, named extract-high-confidence-variants, picks out variants from the GATK and Cortex_var vcf files that are deemed of high confidence. Such variants have each allele represented by at least one sample with no more than 5% reads of the minor allele and (1) read coverage from GATK of between 20x and 1,000x for *M*. *avium* (or between 20x and 2,000x for *M*. *genavense*) or (2) a status of PASS and a genotype confidence score of at least 100 from Cortex_var. The output from this script is another vcf file that includes SNPs as well as insertions and deletions.

The second script, named vcf-to-phylip, generates a multiple sequence alignment in phylip format from the vcf file output by the preceding script considering only isolated SNPs, i.e., ones that are more than 30 bases apart according to GATK or that are not complex SNPs according to Cortex_var and so are separated by at least the k-mer number of bases. The other variants are neglected since they tend to be less reliable. The allele for each sample at an isolated SNP site is that from the variant caller unless the read coverage is less than 20x or greater than 1,000x (or 2,000 for *M*. *genavense*), in which case the allele is specified as unknown, i.e., “-”in the phylip file. Also, if the minor allele frequency of a sample is greater than 5%, the allele is specified as unknown unless the possibility of multiple genotypes within that sample is being considered.

#### GATK selected as the preferred variant caller

The high-confidence SNPs from GATK and Cortex_var were compared for various combinations of samples. For small clusters of closely related samples, the SNPs from the two variant callers are nearly identical. However, as the number of samples increases, Cortex_var tends to lose SNPs found in smaller subsets of samples. Because of this SNP “dropout”, GATK was selected as the preferred variant caller for the results reported here. More information about the comparison of SNPs from GATK and Cortex_var is provided in the Discussion section.

### Resolving multiple genotypes within a single sample

Single birds sometimes harbor multiple genotypes of the same mycobacterial species. If the different genotypes come from different samples taken from the same bird, then resolving those genotypes is straightforward using the scripts described previously. However, the situation is more complex when multiple genotypes are present within a single sample. Thus the vcf-to-phylip script was extended to handle two different genotypes within a single sample using the approach described by Kay, et al., [19] and then was further extended to handle three and even four genotypes in a single sample.

In particular, the extended script computes the minor allele frequency (MAF) at each site with mixed alleles from multiple genotypes and uses that to separate the mixed population into two, three, or four subpopulations with different genotypes as appropriate. Here MAF is defined to be the fraction of the reads with the minor allele or genotype and is required to be greater than some minimum cutoff value to avoid false positives. The resulting subpopulations replace the original sample in the phylip file by multiple subsamples with the ID of the original sample plus decimal suffices.

Note that the only sites considered are those already identified as having a high-confidence SNP, where each allele is represented by at least one sample with no more than 5% reads of the minor allele. This requirement may undercount the number of valid sites with multiple genotypes, but reduces the chance of false positives.

Further discussion of the separation of multiple genotypes within a single sample is deferred to the Results section.

### Clustering samples of the same species

#### Inferring phylogenetic trees

RAxML 8.2.9 (https://github.com/stamatak/standard-RAxML) was run to generate phylogenetic trees from the phylip files containing high-confidence SNPs for the *M*. *avium* and *M*. *genavense* samples. A comprehensive analysis with multiple bootstrap and regular searches was done using the following options: “-m ASC_GTRCAT—asc-corr = lewis–V–N autoMRE–p 12345 –x 12345 –f a”. The options containing “ASC” and “asc” are appropriate for a multiple sequence alignment consisting only of SNPs.

#### Displaying the trees

FigTree 1.4.0 (http://tree.bio.ed.ac.uk/software/figtree) was used to display the trees from RAxML. This allowed visual identification of clusters of samples that are close genomically.

#### Calculating the genetic distance matrix

A custom Perl script, named distance-matrix, calculated the genetic distance matrix between samples within each cluster, starting from the phylip file. This quantified the genomic closeness of the samples within the clusters that were visually identified.

## Results

### Mycobacteria were successfully cultured from 113 birds

These included some of the birds considered previously by Witte, et al. [3], and Schrenzel, et al. [7] Isolates from 45 birds originated from submissions to external laboratories, while in-house procedures were able to successfully culture fresh or frozen tissues from 68 of 122 birds (56%). *M*. *genavense* was isolated from a sample that had been frozen for over 23 years. Overall, 132 tissue samples were cultured from the 113 birds.

### WGS identified 9 mycobacterial species, mostly *M*. *avium* and *M*. *genavense*, in 105 birds

Conventional tests indicated that mycobacteria were present in all of the 132 cultured samples. WGS was thus performed on these samples, and the subsequent analysis with Velvet and BLAST+ showed the presence of mycobacteria in 123 samples from 105 birds. Table 1 shows the concordance in species identification between WGS and the conventional tests.

**Table 1:** Concordance in species identification between WGS and conventional tests for 132 samples.

Concordance	Samples	Species from WGS	Species from conventional tests
**Concordant to species**	1	*M*. *arupense*	*M*. *arupense*
(n = 94; 71%)	33	*M*. *avium*	*M*. *avium*
	1	*M*. *fortuitum*	*M*. *fortuitum*
	58	*M*. *genavense*	*M*. *genavense*
	1	*M*. *intracellulare*	*M*. *intracellulare*
**Concordant to genus**	22	*M*. *avium*	*Mycobacterium* genus
(n = 24; 18%)	1	*M*. *fortuitum*	*Mycobacterium* genus
	1	*M*. *hassiacum + M*. *peregrinum*	*Mycobacterium* genus
**Partially concordant**	1	*M*. *avium* + 2 non-mycobacteria	*Mycobacterium* genus
(n = 2; 2%)	1	*Salmonella enterica*	*M*. *genavense + S*. *enterica*
**Discordant**	1	*M*. *intracellulare*	*M*. *avium*
(n = 12; 9%)	1	*M*. *intracellulare*	*M*. *genavense*
	1	*M*. *URHD0025*	*M*. *avium*
	1	*M*. *vulneris*	*M*. *genavense*
	7	Non-mycobacteria*	*Mycobacterium* genus
	1	Bad reads	*M*. *genavense*

*M. *avium* at very low coverage was found in two of these samples.

For 120 of 132 samples (91%) the species found by WGS and the conventional tests were concordant in full or in part. Included in this category are samples that were conventionally tested only to the genus. The concordance reported here is somewhat lower than the 96% reported by Pankhurst, et al. [20], who looked at 345 samples of mycobacterial species from humans and compared results from WGS and “routine methods”.

Twelve samples (9%) were discordant. WGS is presumed correct for the first four of these samples in Table 1, while WGS is considered to have failed for the last eight samples because of contamination or, in the last case, bad reads. Discordance in mycobacterial species identification seen between conventional tests and WGS may be due to different individual colonies of the same culture being used for mycobacterial confirmatory testing using PCR and subculture for WGS.

The last eight samples in Table 1 plus the sample containing *S*. *enterica* were not further evaluated. This left 123 samples from 105 birds as listed in Table 2, where *M*. *avium* has been separated into the two subspecies found.

**Table 2:** Mycobacterial species found by WGS in 123 samples from 105 birds.

Mycobacterial species	Birds
M. arupense	1
*M. avium avium*	37
*M. avium hominissuis*	12
*M. fortuitum*	2
*M. genavense* only	47
*M. genavense + M. intracellulare*	1
*M. hassiacum + M. peregrinum*	1
*M. intracellulare* only	2
*M. URHD0025*	1
*M. vulneris*	1
Total	105

The most common species were *M*. *avium* and *M*. *genavense*, which were in 49 and 48 birds, respectively. *M*. *intracellulare* and *M*. *fortuitum* were found in three and two birds, respectively, while the other five species were only found in single birds. Two birds had infections from two different mycobacterial species.

The first sheet of S2 File shows that all of the first 123 samples had whole genome sequences that matched an NCBI mycobacterial genome with an ANI ≥ 98% and coverage ≥ 88% (provided that the SPAdes assembly is used for samples with both Velvet and SPAdes assemblies). Thus the species identification seems definitive.

Two samples merit further comment. The first sample, myc28, contains two mycobacterial genomes: *M*. *hassiacum* and *M*. *peregrinum*. The best matching *M*. *peregrinum* genome with an ANI of 98.7% was added to the NCBI database on June 21, 2016, whereas all of the other matching genomes were from a download of the database on March 17, 2016. The second sample, myc11, has a best match to *M*. *URHD0025* in the NCBI database, with an ANI of 98.6%. This species has not been characterized sufficiently to be given a Latin name yet, and no species that have such names have close matches to the sample.

The first sheet of S2 File shows that most *M*. *avium* samples (42 of 56; 75%) have their closest match to *M*. *avium avium ATCC 25291* with ANI ≥ 99.85%. Thus it is reasonable to conclude that these samples are all *M*. *avium avium*. The remaining *M*. *avium* samples have generally lower ANI matches to other strains in the database, but all seem to be *M*. *avium hominissuis*. Their best matches are to strains explicitly listed as *MAH* or to ones for which the nearest matches in the database are *MAH*. An example of the latter is myc34; its closest database match is *MAA DT78*, but that strain most closely matches two *MAH* strains, suggesting that *MAA DT78* may have been mischaracterized as *MAA*. Of further note is that *MAA ATCC 25291* was isolated from the liver of a hen (per http://www.ncbi.nlm.nih.gov/bioproject/PRJNA30909), whereas none of the other matching strains came from birds.

As for the *M*. *genavense* samples, all but one–myc97 –match the single database strain, *M*. *genavense ATCC 51234*, with ANI ≥ 99.97%. This indicates that there is much less genomic variation in the *M*. *genavense* samples than in the *M*. *avium* samples and is consistent with the much greater variability in the *M*. *avium* genome suggested by its large number of strains in the NCBI database.

### Two genotypes of the same species were found in nine samples, and three were found in four samples

Variant calling with GATK and the subsequent filtering for high-confidence SNPs revealed that some samples containing *M*. *avium* and *M*. *genavense* had multiple genotypes. This was apparent from the presence of many sites with mixed alleles and well-defined peaks in the frequency distribution of MAF. The first sheet of S3 File provides MAF summary information for the single *M*. *avium* sample and 12 *M*. *genavense* samples that clearly had multiple genotypes, while the remaining sheets show tables and plots of the MAF distributions for these samples. Additional samples likely had multiple genotypes, but their MAF distributions contained relatively few sites or had peaks below the minimum MAF cutoff and so were not considered.

The number of genotypes present in a sample can be determined from the number of peaks in the MAF distribution. All but four of the samples with multiple genotypes have a single peak in the MAF distribution below 50% MAF, which separates the major and minor alleles. These samples have two genotypes. For most of these samples the distribution is well separated from the 50% maximum MAF and the minimum MAF cutoff, which was taken to be 20% for *M*. *avium* and 6% for *M*. *genavense*. For example, the data sheet and plot in S3 File for myc91 show a peak in the distribution at 24% MAF with the two genotypes separated by 85 SNPs, the number of sites with mixed alleles. The first diagram in S4 File illustrates this case. The percentages shown correspond to the proportion of reads (and presumably cells) in the sample with each genotype based on the MAF at the peak.

Three multi-genotype samples–myc21, myc106, and myc127 –have MAF distributions that overlap 50% MAF. In this case, some SNPs with MAF values less than 50% might actually have the genotype of the major allele because of uncertainty in the MAF measurements. This ambiguity is neglected for myc21 and myc127. However, for myc106, the read counts at two sites above 48% MAF were adjusted in the vcf file from the extract-high-confidence-variants script to change the allele at those sites to have the same reference minor allele and genotype as the other sites in myc106.2. These adjusted sites are highlighted in blue in the corresponding data sheet of S3 File. This adjustment allows myc106.1 to be genomically identical to myc113.1, which is reasonable since the two samples came from the same bird.

Two samples–myc71 and myc106 –have two peaks in the MAF distribution per the plots in S3 File. These samples have three genotypes. Diagrams in S4 File show the two possible three-genotype trees consistent with these distributions and, in each case, note the one that seems more likely. For myc71, the chosen three-genotype tree minimizes the distance of myc71.3 from the other *M*. *genavense* samples. For myc106, the two outlier sites with less than 7% MAF are assumed not to be mixed. This allows myc106.3 for the chosen three-genotype tree to be genomically identical to myc68.1 and myc107.1.

One sample–myc107 –has three peaks in the MAF distribution below 50% MAF. This distribution could arise from a three-genotype tree or five possible four-genotype trees. The last diagram in S4 File shows the three-genotype tree that seems most likely and that was used in the subsequent analysis. In this case, the genotype denoted by myc107.2 is artificial, as noted by 0% cells in the diagram. myc107.2 corresponds to the SNPs relative to myc107.1 that are shared by myc107.3 and myc107.4 and that cause a peak in the MAF distribution at 34%.

### Multiple genotypes of the same species were found in 4 of 49 birds (8%) with *M*. *avium* and 11 of 48 birds (23%) with *M*. *genavense*

Multiple genotypes per bird were sometimes found not only within single samples, as just discussed, but also within repeat samples from the same bird. Table 3 provides a breakdown of the number of genotypes per bird considering both single and repeat samples. One bird with *M*. *avium* and three with *M*. *genavense* had three distinct genotypes, and one bird with *M*. *genavense* had four distinct genotypes.

**Table 3:** Number of genotypes per bird showing the presence of mixed infections and within-host evolution.

Mycobacterial species	Genotypes per bird	Birds with one sample	Birds with two samples from two tissues	Birds with three samples from two tissues	Total birds
***M. avium***	One	42	3	0	45
	Two different by ≤ 5 SNPs	0	2	0	2
	Two different by > 12 SNPs	1	0	0	1
	Three different by > 12 SNPs	0	0	1	1
	Total	43	5	1	49
***M. genavense***	One	29	8	0	37
	Two different by > 12 SNPs	7	0	0	7
	Three different by ≥ 10 SNPs	2	1	0	3
	Four different by > 12 SNPs	0	1	0	1
	Total	38	10	0	48

Twelve of the 15 birds with multiple genotypes had ones that were separated by more than 12 SNPs, the maximum number suggested by Walker, et al., [8] for within-host evolution. These appear to have been the result of mixed infections. Two birds had two genotypes separated by ≤ 5 SNPs. These are likely the result of within-host evolution. The one remaining bird had three genotypes; two were 10 SNPs apart and may have been from within-host evolution, while the third genotype was tens of SNPs apart from the others and was the result of mixed infection.

### The *M*. *avium* samples represent diverse strains

GATK analyses were done to find the SNPs between samples for each species that was present in more than one bird, namely *M*. *avium*, *M fortuitum*, *M*. *genavense*, and *M*. *intracellulare*. Genomic clusters of samples with genotypes that differed by relatively few SNPs were found for *M*. *avium* and *M*. *genavense*, but not for *M*. *fortuitum* or *M*. *intracellulare*.

**Fig 1:**
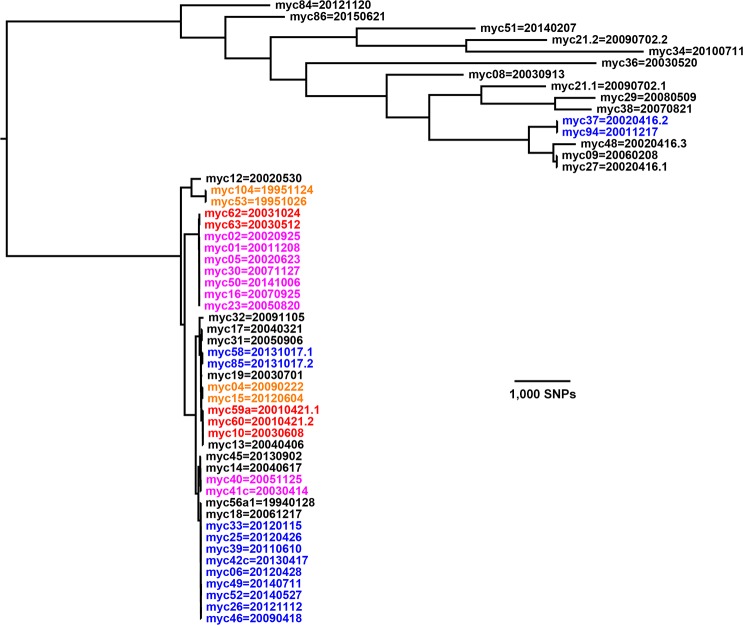
Tree for 53 *M*. *avium* samples. Each sample or subsample in one of the colored clusters is ≤ 12 SNPs from at least one other sample in the cluster. The two clades above and below the arbitrary root have very different shapes. The upper clade is spread out with some samples separated from each other by nearly 10,000 SNPs. By contrast, the separation between the samples in the lower clade is much less and is better resolved in Fig 2. Based on matches to the NCBI database, the 39 samples in the lower clade are all *M*. *avium avium*, while the 14 samples in the upper clade all seem to be *M*. *avium hominissuis*.

Fig 1 shows the phylogenetic tree from RAxML for 53 samples containing *M*. *avium*. Included are all but the three duplicate samples listed in the “Repeat genotypes” sheet of S2 File. Each leaf on the tree is labeled by both the sample and bird IDs.

### Samples from 31 of 49 birds with *M*. *avium* are in nine clusters with closely related genotypes

**Fig 2:**
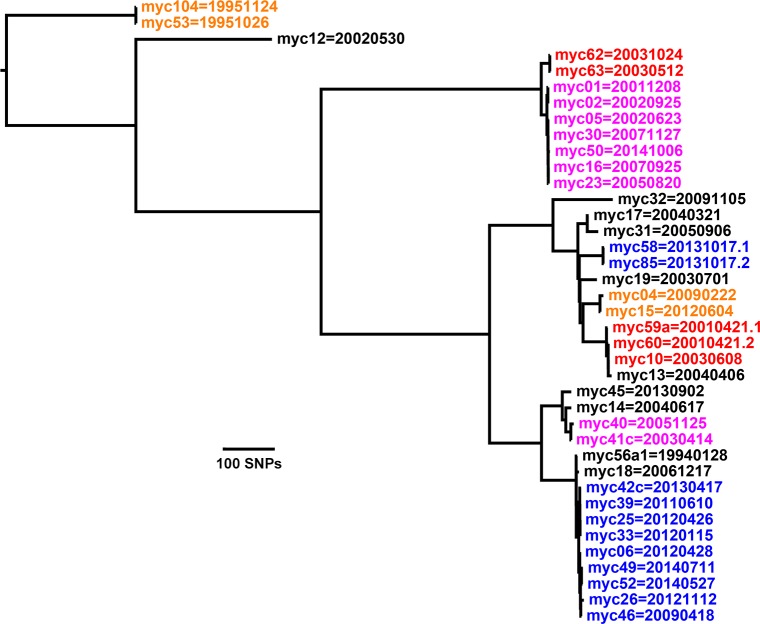
Tree for 39 *M*. *avium avium* samples. Each sample or subsample in one of the colored clusters is ≤ 12 SNPs from at least one other sample in the cluster. Although not shown on the trees, the bootstrap support values are 100% at all of the nodes outside of the colored clusters. Thus the overall tree topology is very robust.

Each of these clusters is highlighted by color in Figs 1 and 2. Six clusters have only two samples. Each sample in one of these clusters is ≤ 12 SNPs from at least one other sample in the cluster. The choice of 12 SNPs for the cluster threshold and the genotype categories in Table 3 and the figures is suggested by the transmission analysis of *M*. *tuberculosis* reported by Walker, et al. [8], who stated: “We expected epidemiological linkage consistent with transmission to exist between isolates differing by five or fewer SNPs, and not to exist between isolates differing by more than 12 SNPs. We deemed pairs differing by six to 12 SNPs to be indeterminate.” Adopting the same SNP thresholds for *M*. *avium* and *M*. *genavense* seems reasonable for studying transmission and within-host evolution provided that their mutation rates are similar to that of *M*. *tuberculosis*. For the study here, which is not analyzing transmission, the 12-SNP threshold provides a convenient way to visualize genomic closeness within the obvious clusters in Figs 1 and 2.

The upper clade in Fig 1 includes just one colored cluster; it has two samples–myc37 and myc94 –which differ by 10 SNPs. The next closest pair of samples in that clade consists of myc09 and myc27, which differ by 23 SNPs.

Two sets of samples in the upper clade of Fig 1 are from two birds with mixed infections. Sample myc21 from Bird 20090702 contains two genotypes that are separated by thousands of SNPs. The two genotypes are denoted by decimal suffices on the IDs for the sample and associated bird. The remaining mixed infection is from Bird 20020416 and is in three separate samples, which differ from each other by hundreds to thousands of SNPs.

The lower clade in Fig 1, as well as its better-resolved version in Fig 2, includes eight colored clusters. The genetic distance matrices for these clusters are in the “12apart M. avium” sheet in S2 File. Three clusters contain more than two samples. Although the minimum pairwise distance for each sample in one of these clusters is ≤ 12 SNPs, the maximum pairwise distance is larger: 15 SNPs in the 7-sample cluster containing myc01, 31 SNPs in the 9-sample cluster containing myc06, and 22 SNPs in the 4-sample cluster containing myc10. Moreover, three more samples–myc13, myc18, and myc56a1 –would fit in one of the latter two clusters if the threshold were increased to 13 SNPs or if Cortex_var were used instead of GATK to call the SNPs. This suggests that a slightly larger threshold might be more appropriate for transmission analysis of *M*. *avium*. The next closest pair of samples not in a cluster consists of myc17 and myc31, which are 19 SNPs apart.

S2 File also contains a “5apart M. avium” sheet with genetic distances for clusters within which the minimum pairwise separation for each sample is ≤ 5 SNPs. Such clusters correspond to the more stringent threshold given by Walker, et al.

### The *M*. *genavense* samples tend to be closer to each other genomically than the *M*. *avium* samples are

**Fig 3:**
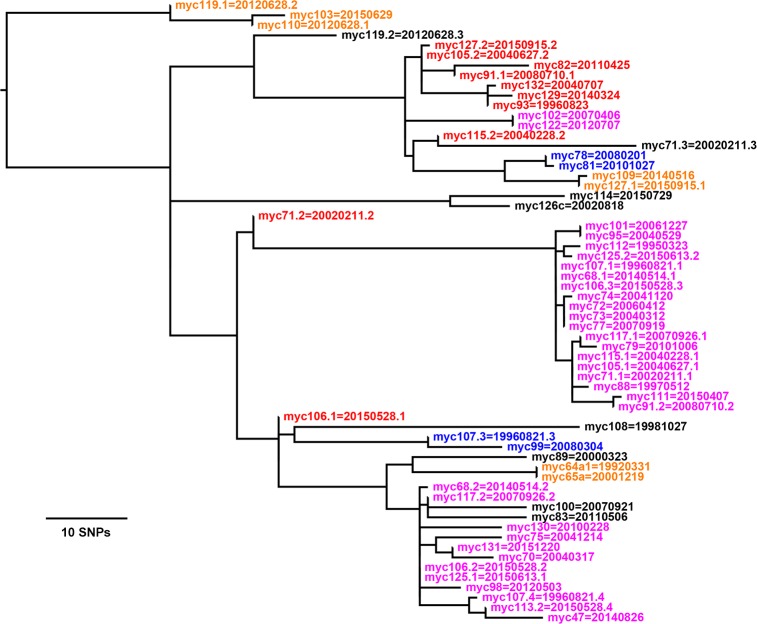
Tree for 46 *M*. *genavense* samples. Each sample or subsample in one of the colored clusters is ≤ 12 SNPs from at least one other sample in the cluster. Although not shown on the tree, the bootstrap support values are between 97 and 100% for most but not all of the nodes outside of the colored clusters. Thus the overall tree topology is not quite as robust as that for the *M*. *avium* trees, presumably because the relevant branch lengths measured in SNPs are much shorter in the *M*. *genavense* tree.

Fig 3 shows the phylogenetic tree for samples from all but two of the 48 birds with *M*. *genavense*. The genetic distances between samples in the tree are generally much shorter than in the trees for *M*. *avium*. The largest genetic distance between any pair of samples shown is less than 110 SNPs. The omitted samples, one per bird, are myc97 and myc128, which are separated by hundreds to thousands of SNPs from the other samples. Also omitted are eight duplicate samples and one duplicate subsample listed in the “Repeat genotypes” sheet of S2 File.

### Samples from 40 of 48 birds with *M*. *genavense* are in 10 clusters with closely related genotypes

Many of the *M*. *genavense* samples contained multiple genotypes. This and their genomic closeness made identification of their clusters more complex than for the *M*. *avium* samples.

The clusters where pairs of samples differ by ≤ 12 SNPs are highlighted by color in Fig 3. The largest cluster with myc68.1 contains 19 samples, and the maximum pairwise distance within that cluster is only 11 SNPs, as can be seen in the “12apart M. genavense” sheet in S2 File. This same cluster also appears in the “5apart M. genavense” sheet in S2 File. The other two large clusters–one with myc47 and the other with myc82 –are more spread out, with maximum pairwise distances of 25 SNPs and 24 SNPs, respectively.

Some birds have samples in more than one cluster because of the mixed infections. Only 8 of 48 birds with *M*. *genavense* do not have a sample in one of the clusters.

## Discussion

### Need to culture mycobacteria to obtain high read coverage

The need to culture mycobacteria from tissues to obtain enough DNA for high read coverage during WGS is a noteworthy limitation for the SNP analysis. In particular, identifying a SNP with high confidence using the filters adopted here requires that there be enough mycobacterial DNA in the sample for the read coverage to be at least 20x at the SNP site. To achieve this at nearly all sites in the genome, the average filtered read coverage must be about 50x or more. The first sheet of S2 File shows that this was achieved for all but four samples: myc03, myc38, myc44c, and myc124.

For myc38 the average filtered read coverage was 33x, and the local coverage dropped below 20x at many SNP sites resulting in unknown alleles there. Nonetheless, this coverage was sufficient to achieve a good match to *M*. *avium MAV_061107_1842* across 96% of its genome and to show that the sample was not genomically close to any others. For myc03 and myc44c the average read coverage was even lower, resulting in good matches to *MAA ATCC 25291* across 16% and 4% of its genome, respectively. This coverage was insufficient to identify any high-confidence SNPs. For myc124 the reads were bad and could not be further analyzed.

Because of the difficulty in culturing mycobacteria from tissue samples, an attempt was made to enzymatically enrich mycobacterial DNA extracted from three tissue samples without culturing. WGS was then done on the extracted DNA. For two of these samples the coverage was sufficient to achieve a good match to *M*. *avium* across 11% and 13% of its genome, similar to that for myc03, but was insufficient to identify any high-confidence SNPs. For the third sample, no match to a mycobacterial genome was found.

Nonetheless, it seems likely that WGS will eventually work reliably with sufficiently small amounts of DNA that culturing will not be necessary. When this happens, the use of WGS should become routine for investigating mycobacteriosis.

### Comparison of variant-calling approaches

The high-confidence SNPs from GATK and Cortex_var are nearly identical for small clusters of closely related samples. This can be seen from the 5apart and 12apart distance matrices for *M*. *avium* in S2 File. In particular, the 5apart matrices are the same for four of seven clusters and differ by only one or two SNPs for the other three clusters.

As the number of samples increases, however, Cortex_var tends to lose SNPs found in smaller subsets of samples, whereas GATK loses very few. An example of this SNP dropout can be seen already in going from the 5apart to the 12apart matrices from Cortex_var where the two SNPs that separate myc01 and myc02 in the smaller 2x2 matrix disappear in the larger 7x7 matrix. For the 39 *M*. *avium* samples shown in Fig 2, which corresponds to a 39x39 matrix, Cortex_var finds only half as many SNPs as does GATK. The reason for the SNP dropout in Cortex_var is unknown. For GATK the small dropout arises because some SNPs become closer together than the 30-base distance allowed by the vcf-to-phylip filter to ensure that the SNPs remain valid. In light of the preceding, GATK was selected as the preferred variant caller for the results reported here.

The GATK best practices recommend that variants be called in two steps, with HaplotypeCaller invoked for each sample to generate corresponding gvcf files, which are then input to GenotypeGVCFs to obtain a single vcf file for all samples. However, this two-step approach was found to overlook some valid SNPs. Thus HaplotypeCaller was invoked instead to generate the final vcf file in a single step, which sometimes gave a larger number of valid SNPs. The downside of the single-step approach is that the run time increases substantially with the number of samples. The HaplotypeCaller run to generate the SNPs for the 53 *M*. *avium* samples shown in Fig 1 took six days on a 16-core node of the Gordon supercomputer at the San Diego Supercomputer Center. However, the run times for the smaller clusters, which are of most interest for transmission studies, were only several hours.

### Multiple genotypes from mixed infections and within-host evolution

Single individuals with mycobacteriosis sometimes harbor multiple genotypes of the same mycobacterial species. Such within-host diversity can arise from a mixed infection, in which an individual is infected by multiple strains, or from microevolution within the host following a single infection. Cohen, et al. [21], reviewed many studies of mixed infections of *M*. *tuberculosis* found by conventional tests, while Hatherell, et al. [22], reviewed more recent studies of mixed infections and within-host evolution of *M*. *tuberculosis* found by WGS. Examples of both mixed infections and within-host evolution were found in the *M*. *avium* and *M*. *genavense* samples considered here.

Multi-genotype *M*. *tuberculosis* infections, especially mixed ones, often occur in humans, though their reported prevalence based on conventional methods of detection varies widely as summarized in Table 1 of Cohen, et al. [21] For example, mixed infections were found in 35 of 186 patients (19%) by Warren, et al. [23], but in only 2 of 97 patients (2%) by Shamputa, et al. [24] The varying percentages are likely due to environmental differences as well as limitations in detection. In addition, definitions of mixed infections and within-host evolution vary [22]. In another noteworthy study, Kay, et al. [19], used WGS to find mixed infections in 5 of 8 bodies (63%) of people who died in Hungary about 200 years ago and concluded that such infections were more prevalent then. Four distinct genotypes in single patients were reported in two studies [24, 25].

Two studies of *M*. *tuberculosis* by Shamputa, et al. [24, 26], found both mixed infections and within-host evolution in the study cohorts. The first study of 97 patients used DNA fingerprinting to find mixed infections and within-host evolution in 2 and 8 patients (2% and 8%), respectively, while the second study of 199 patients used PCR typing to find the same two classes of infection in 26 and 7 patients (13% and 4%), respectively. The thresholds for calling a mixed infection between two genotypes were differences by “more than three IS6110-RFLP bands” for DNA fingerprinting [24] and “detection of double alleles in multiple loci” for PCR typing [26]. The latter test provides higher resolution in detecting mixed infections, which is part of the reason for their higher percentage in the second study.

More recently, Pérez-Lago, et al. [13], used WGS to investigate within-host evolution of *M*. *tuberculosis* in four patients. They looked at multiple samples from the same or different tissues collected within a few days of each other and found differences of between 3 and 8 SNPs in each patient. In addition, Lieberman, et al. [14], performed WGS on 2,693 samples collected postmortem from 329 sites across 44 patients infected by *M*. *tuberculosis*. They found mixed infections in 4 patients (9%) and within-host evolution in 34 patients (77%). Of the latter, five patients had differences > 5 SNPs, including one patient with a maximum difference of 14 SNPs.

Multi-genotype, mixed infections have also been reported for *M*. *avium* based on conventional tests. In particular, Arbeit, et al. [27], used PFGE to find two genotypes of *M*. *avium* in blood from 2 of 14 human patients (14%); Shitaye, et al. [6], used RFLP to find two genotypes of *MAA* in tissues from 7 of 16 infected hens (44%); and Dvorska, et al. [5], used RFLP to find *MAH* in tissues from 9 of 19 water birds (47%) infected by *MAA*, On the other hand, multi-genotype infections do not seem to have been reported before for *M*. *genavense*.

By comparison, the current study found multi-genotype infections from *M*. *avium* in 4 of 49 birds (8%) and from *M*. *genavense* in 11 of 48 birds (23%). Most of these were mixed infections. However, two birds each had two *M*. *avium* genotypes apparently from within-host evolution, and one bird had three *M*. *genavense* genotypes that appeared to be from both a mixed infection and within-host evolution. Four distinct *M*. *genavense* genotypes were found in one bird. Additional cases of multiple genotypes may have been present in single samples, but are not easily distinguished from errors in the data or analysis.

Shamputa, et al. [26], found that the likelihood of detecting mixed *M*. *tuberculosis* infections increased as more samples from the same patient were collected and concluded that mixed infections are often overlooked and underestimated. This is consistent with the results reported for *M*. *avium* in Table 3, where multi-genotype infections were found in only 1 of 43 birds with a single sample, but in 3 of 6 birds with multiple samples. On the other hand, the results for *M*. *genavense* in Table 3 show multiple-genotype infections in 9 of 38 birds with single samples, but in only 2 of 10 birds with multiple samples. Nonetheless, the many multiple-genotype infections of *M*. *avium* and *M*. *genavense* found here in birds and the few reports by others suggest that such infections are also overlooked and underestimated in zoo populations. This is noteworthy because the presence of such infections greatly complicates efforts to unravel transmission dynamics [28].

### Mutation rate of mycobacteria

Several researchers have reported estimates for the mutation rate of *M*. *tuberculosis*. For example, Walker, et al., [8] obtained an estimate based upon repeat measurements of SNPs at different times and inferred a mutation rate of 0.5 SNP per genome per year. Additional mutation-rate estimates of 0.4 and 0.3 SNP per genome per year were obtained by Roetzer, et al., [11] and Bryant, et al., [12] respectively.

By contrast, the mutation rate does not seem to have been measured previously for either *M*. *avium* or *M*. *genavense*. To obtain an estimate for *M*. *avium*, two of the previous samples–myc01 and myc25 –were cultured for two years, and their DNA was periodically extracted and sequenced. During this time, four SNPs occurred and became fixed: one from myc25 after six months and three from myc01 only after the full two years. This gives an estimated mutation rate of one SNP per genome per year. Given the small number of measured events, this mutation rate estimate for *M*. *avium* is consistent with the very low estimates reported for *M*. *tuberculosis*.

## Conclusions

The study reported here is the largest to evaluate genetic relatedness between mycobacterial strains isolated from birds in a single population over a long time period and the first to do so using WGS. Several advantages of WGS compared to conventional tests are noteworthy.

1. WGS provided more definitive species identification. One sample contained two mycobacterial species, where only one was found by conventional tests, while another sample contained an uncommon species not identified by conventional tests.
2. WGS allowed multiple genotypes of the same species to be resolved in single samples. In particular, two genotypes of the same mycobacterial species were found in nine samples, and three were found in four samples.
3. WGS clearly showed that the *M*. *avium* samples were more diverse genomically than were the *M*. *genavense* samples.
4. By resolving samples to individual SNPs, WGS identified nine genomic clusters for *M*. *avium* and ten for *M*. *genavense* within which any sample is ≤ 12 SNPs from at least one other sample in the cluster.

Knowledge of such genomic clusters is necessary but not sufficient to infer mycobacterial transmission based on epidemiological links among the host birds. The San Diego Zoo and its Safari Park house over 3,000 birds at a given time, and these are typically moved between enclosures several times in their lifetime for breeding, behavioral, or management purposes. Contact tracing using housing history records linked to the time spent together in a shared environment is complex and requires the development of additional methodology. That is the subject of an ongoing, companion study on transmission dynamics in which the genomic clusters of mycobacteria are being correlated with the spatiotemporal clusters of birds.

## Supporting information

S1 File Information on sequencing of samples and parameters used in analysis. (XLSX)

S2 File Information on results of assemblies, alignments, and clusters of samples by bird. (XLSX)

S3 File Information on infections with multiple genotypes. (XLSX)

S4 File Diagrams showing relationship of genotypes within samples containing mixed infections. (PPT)
