# Supplementary material for: Whole-genome analysis of mycobacteria from birds at the San Diego Zoo
Source: PLoS One. 2017 Mar 7;12(3):e0173464. doi: 10.1371/journal.pone.0173464 (PMC5340394; doi:10.1371/journal.pone.0173464)
Supplement: S4 File — (PPT) [file pone.0173464.s004.ppt]

## Slide 1
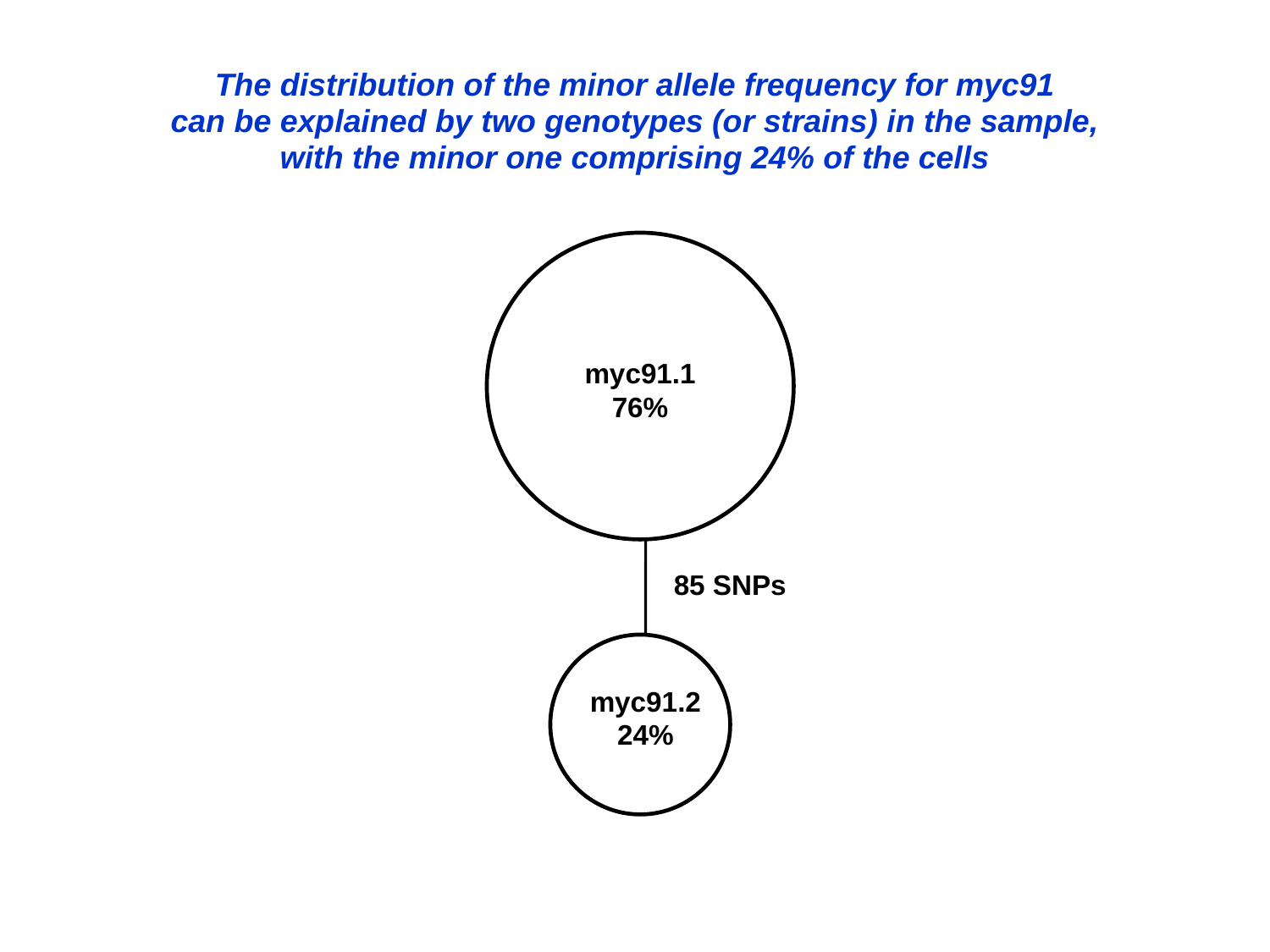

# The distribution of the minor allele frequency for myc91 can be explained by two genotypes (or strains) in the sample, with the minor one comprising 24% of the cells
myc91.1
76%
85 SNPs
myc91.2
24%

## Slide 2
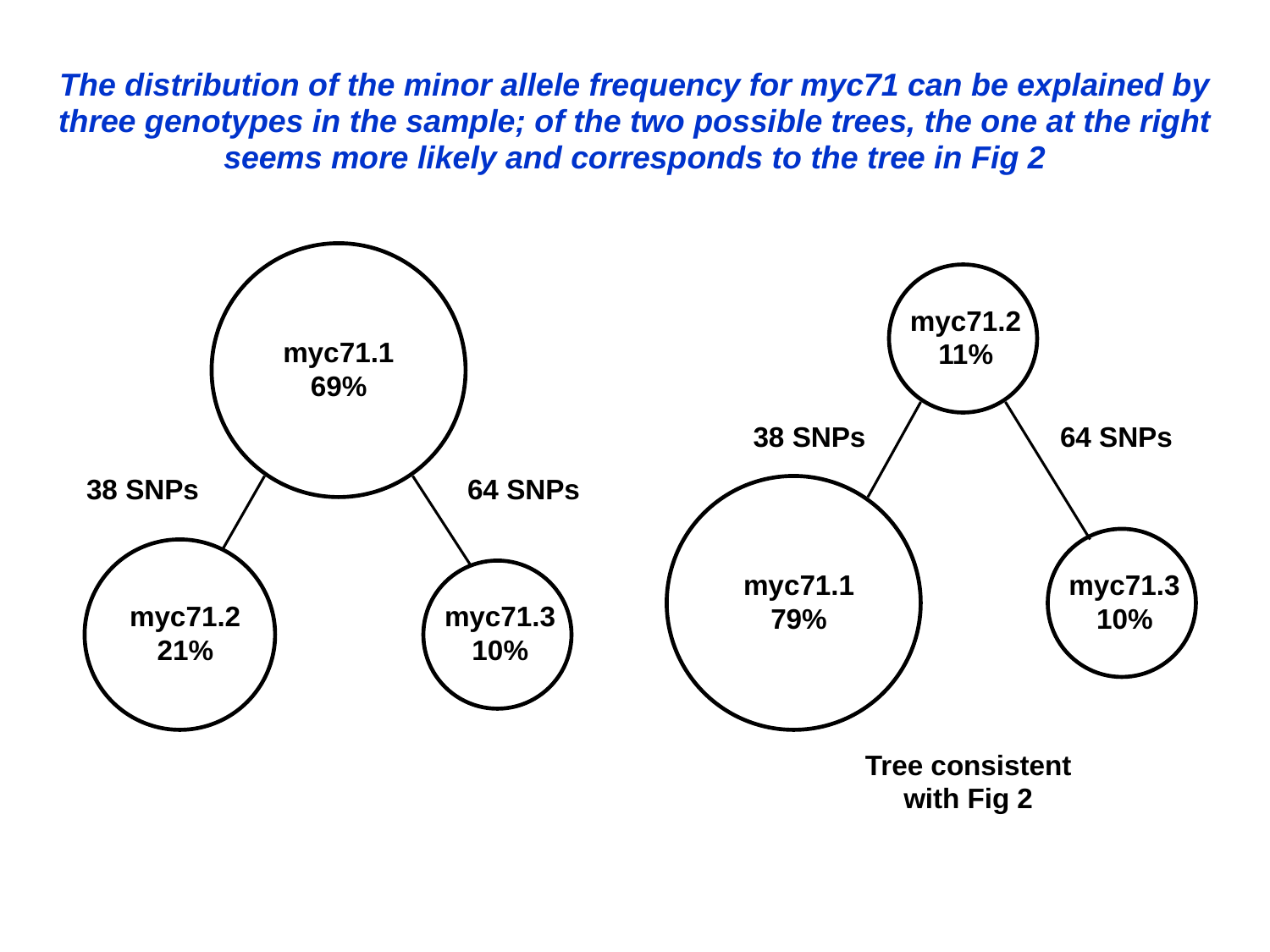

# The distribution of the minor allele frequency for myc71 can be explained by three genotypes in the sample; of the two possible trees, the one at the right seems more likely and corresponds to the tree in Fig 2
myc71.2 11%
myc71.1
69%
38 SNPs
64 SNPs
38 SNPs
64 SNPs
myc71.1 79%
myc71.3 10%
myc71.2 21%
myc71.3 10%
Tree consistent
with Fig 2

## Slide 3
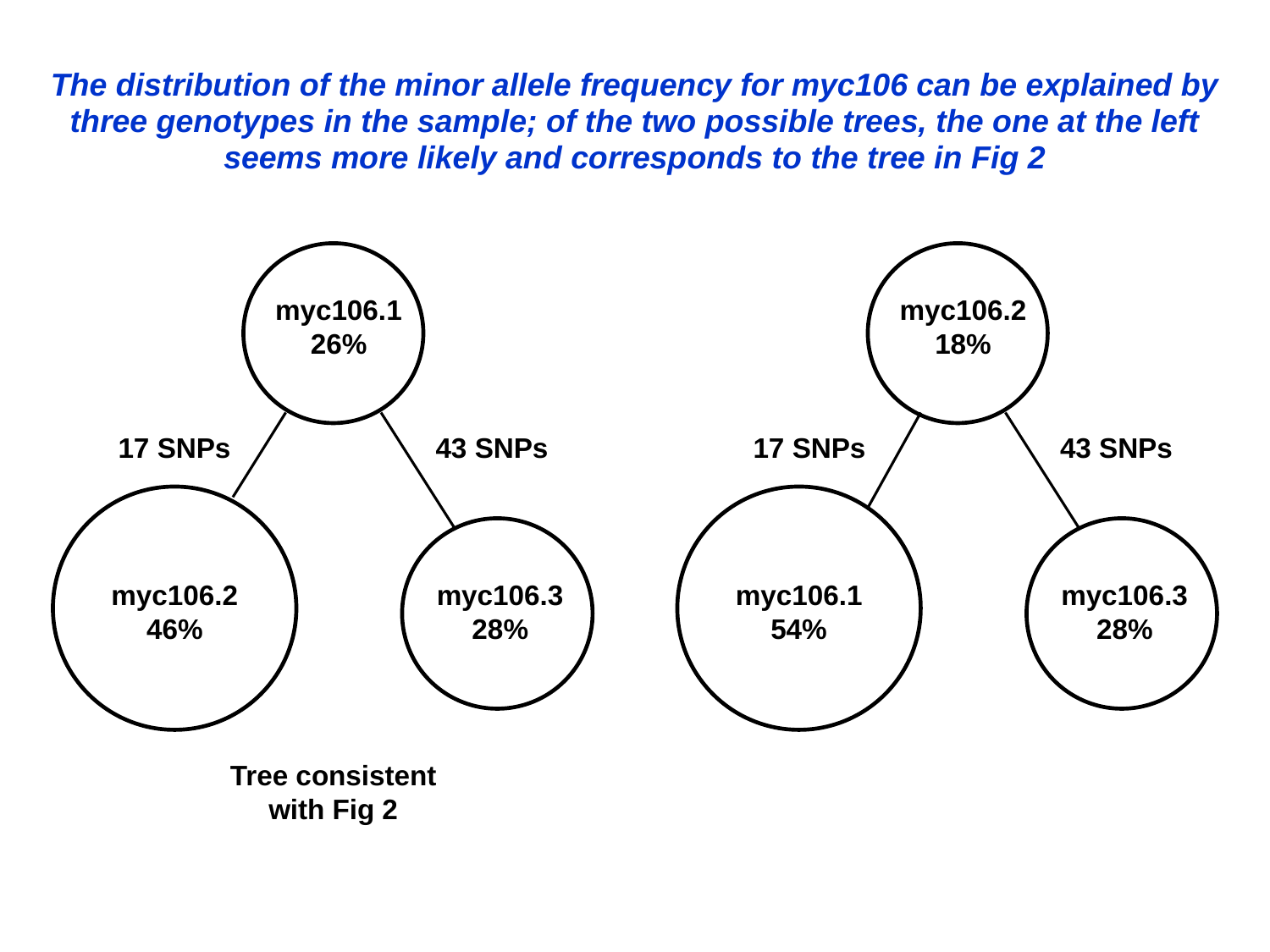

# The distribution of the minor allele frequency for myc106 can be explained by three genotypes in the sample; of the two possible trees, the one at the left seems more likely and corresponds to the tree in Fig 2
myc106.1
26%
myc106.2
18%
17 SNPs
43 SNPs
17 SNPs
43 SNPs
myc106.2 46%
myc106.3 28%
myc106.1 54%
myc106.3 28%
Tree consistent
with Fig 2

## Slide 4
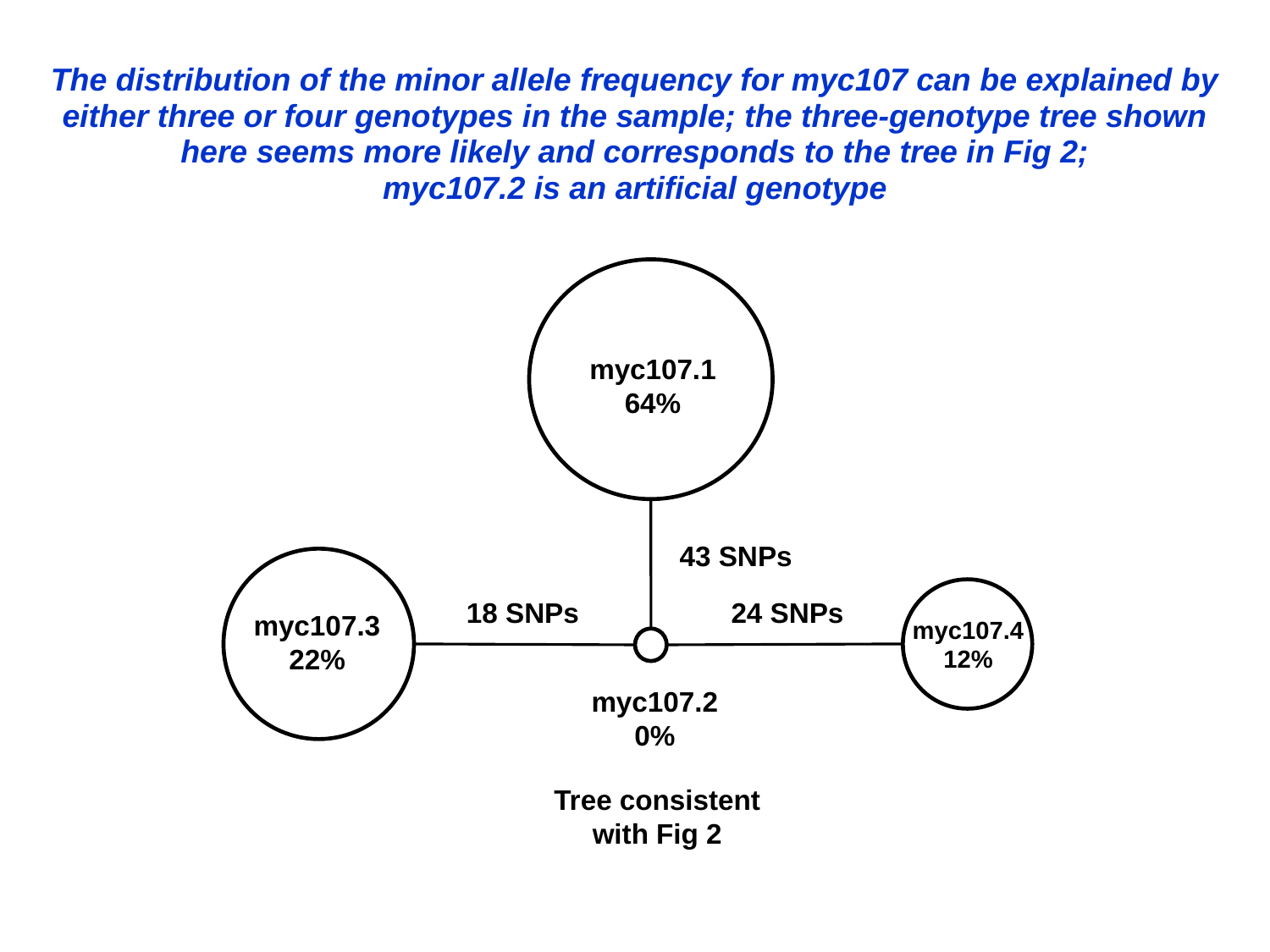

# The distribution of the minor allele frequency for myc107 can be explained by either three or four genotypes in the sample; the three-genotype tree shown here seems more likely and corresponds to the tree in Fig 2; myc107.2 is an artificial genotype
myc107.1
64%
43 SNPs
24 SNPs
18 SNPs
myc107.3 22%
myc107.4 12%
myc107.2 0%
Tree consistent
with Fig 2
